# Ulcerative Granular Cell Tumor: A Clinicopathological and Immunohistochemical Study

**DOI:** 10.1155/2011/497648

**Published:** 2011-11-01

**Authors:** Mohamed El-Khalawany, Al-Sadat Mosbeh, Fatma Abd-Al Salam, Amany Abou-Bakr

**Affiliations:** ^1^Department of Dermatology, Al-Hussein University Hospital, Al-Azhar University, Box 32515, Al-Darasah, Cairo, Egypt; ^2^Department of Dermatology, Al-Zahraa University Hospital, Al-Azhar University, Cairo, Egypt; ^3^Department of Pathology, National Cancer Institute, Cairo University, Cairo, Egypt

## Abstract

Granular cell tumor (GCT) is uncommonly presented with cutaneous ulcer. We examined the clinicopathological and immunohistochemical features of this ulcerative form in fourteen cases that may raise the awareness of this variant. The study included 11 males and 3 females with a mean age 31.5 ± 7.42 years. All cases were presented with large solitary ulcer with indurated base, elevated border, skin colored margin, and necrotic floor. Twelve lesions were located on the extremities and two lesions on the genital region. Histologically, the lesions showed dermal infiltrate composed of large polygonal cells with granular cytoplasm and characteristic infiltration of the dermal muscles in all cases. Immunostaining showed positive reaction for S100 (14/14), NSE (14/14), CD68 (5/14), and Vimentin (7/14) while HMB45, CK, EMA, and Desmin were negative. We hope that this paper increases the awareness of ulcerative GCT and consider it in the differential diagnosis of ulcerative lesions.

## 1. Introduction

Granular cell tumor (GCT) is an uncommon condition of the skin that was described firstly by Weber in 1854 and established as a clinical entity by Abrikossoff in 1926 who termed it as granular cell myoblastoma [1]. The tumor occurs frequently among women and blacks, between the second and sixth decades of life. The common location of GCT is the oral cavity, but it can also occur at any other sites. Cutaneous lesions constitute about 30% of cases; only 1 to 3% is malignant [2].

GCT of the skin is commonly presented with asymptomatic, slow-growing solitary nodule with overlying normal skin. Multiple GCT was also reported as unusual presentation [3], and malignant transformation is considered in lesions that rapidly grows or invades the adjacent tissues [4]. 

The characteristic histological feature of GCT is the coarse eosinophilic cytoplasmic granules which represent lysosomes similar to that found within Schwann cells when ingest myelin [5]. Although GCT was suggested firstly to originate from myoblasts, it is accepted now that other cells such as histiocytes, fibroblasts, undifferentiated mesenchymal cells, and Schwann cells are implicated in the histogenesis [6]. 

Secondary ulceration is uncommon in GCT, and to our knowledge there was no previous study that fully discussed the criteria of this clinical variant. In this study, we highlight the clinicopathological and immunohistochemical features of this ulcerative variant that help to distinguish it from other common ulcerative lesions.

## 2. Materials and Methods 

A total of fourteen cases were enrolled in this study, and they were collected from Al-Azhar university hospitals and the National Cancer Institute, Cairo, Egypt during the period from 2000 to 2010. Clinical data including age, sex, onset, course, and duration of the lesion in addition to the clinical characteristics of the ulcer (size, location, morphology, base, surface, margin, and border) were recorded for each case. History of similar previous lesions or other chronic ulcer(s) in addition to history of other dermatologic or systemic disorders was also recorded. Medical photography and skin biopsy were performed after written consent from each patient. 

A skin biopsy was obtained from the edge of the ulcer, and the specimen was preserved in formalin and embedded in paraffin for processing. Routine hematoxylin and eosin, special staining with periodic acid-Schiff (PAS) and Masson trichrome, immunohistochemical staining with S-100 protein, neuron specific enolase (NSE), cytokeratin (CK), CD68, HMB-45, epithelial membrane antigen (EMA), Vimentin, and Desmin were done for each case.

Immunohistochemical staining was performed using avidin-biotin peroxidase complex method on formalin-fixed, paraffin-embedded tissue sections [7], with a 1/50 dilution of monoclonal antibodies (*Dako, Denmark*). Briefly, tissue sections were mounted on 3-aminopropyl-triethoxysilane-coated slides and dried overnight at room temperature. Subsequently, they were dewaxed in xylene and rehydrated in graded ethanol. After being rinsed with phosphate-buffered saline, they were immersed in 0.01 mol/L citric acid titrated to pH 6.0 and heated twice for 10 minutes in a microwave oven. The primary antibodies were then incubated on the sections for 30 minutes. Diaminobenzidine was used as a chromagen, and the slides were counterstained with Mayer's hematoxylin.

## 3. Results 

Out of 117 cases of cutaneous GCT, only 14 cases (12%) were presented with cutaneous ulcer during the time of diagnosis. The study included 11 males and 3 females with male to female ratio 3.6 : 1. The age of patients ranged from 17 to 42 years (mean 31.5 ± 7.42). The tumor was appeared as asymptomatic nodular lesion with normal overlying skin which was slowly progressed and gradually ulcerated. The duration of the lesion ranged from 23 to 51 months (mean 37.2 ± 8.32) while the time of secondary ulceration ranged from 19–41 months (mean 31.5 ± 6.71).

**Figure 1:**
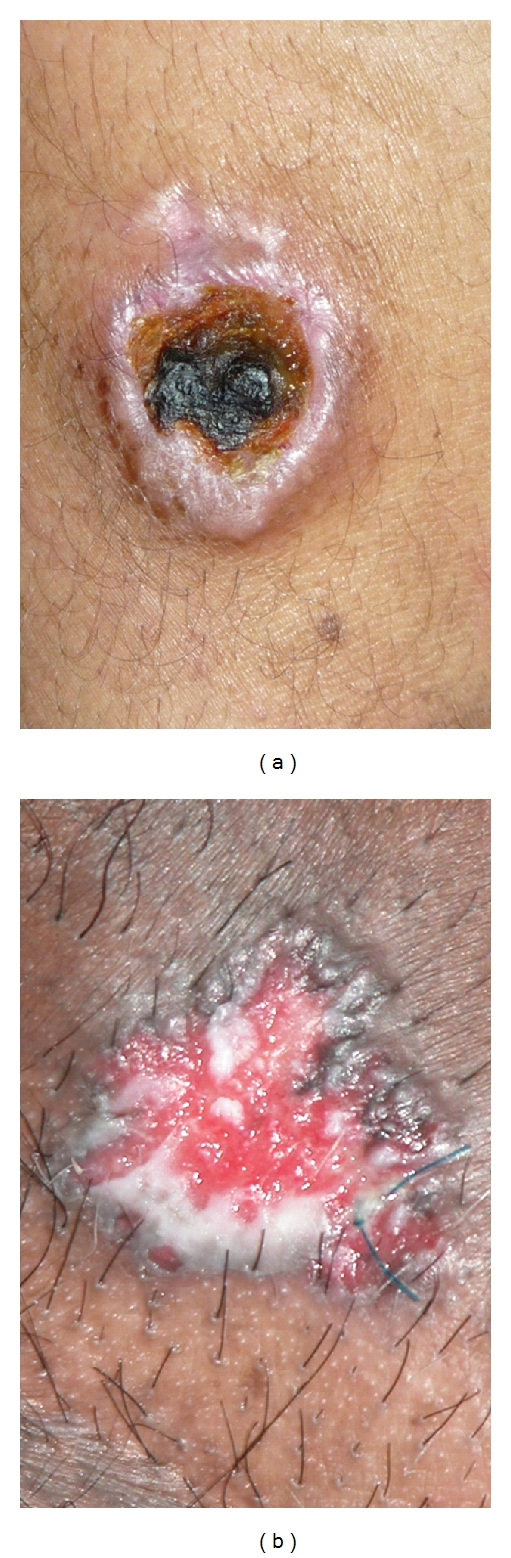
GCT presented with a solitary large ulcerative lesion on the arm (a) and scrotum (b).

At the time of diagnosis, all cases were presented with solitary large rounded or oval ulcerative lesion; twelve were located on the extremities (5 arm, 2 forearm, 3 foot, and one lesion on both leg and thigh) while two lesions were located on the anogenital region (scrotum and perineum). The size of ulcers ranged from 3.8 × 3.5 cm to 5.2 × 5.1 cm with average 4.1 × 3.9 cm. The base was indurated with a characteristic firm to hard consistency (button like) and extended beyond the surface (ranged from 0.7 to 1.2 cm). The floor of ulcer was dry and filled with clean necrotic tissue in the acral lesions (Figure 1(a)), while in the anogenital lesions; it was filled with granulation tissue and discharging minimal exudates (Figure 1(b)). The border of ulcers was slightly elevated while the margin showed normal skin. There was no tenderness of any lesion, and lymph nodes were mildly enlarged in anogenital lesions without associated symptoms while in acral lesions there were no abnormal changes in the regional lymph nodes. 

The clinical diagnosis in 9 lesions was suspected as an infectious granuloma (leishmaniasis, lupus vulgaris, bilharziasis, and chancroid) while in 5 lesions, a malignant neoplasm (squamous cell carcinoma, malignant lymphoma, malignant melanoma, and soft tissue tumor) was suspected. There was no reported past history of similar lesion or other skin disorder at the same site of the lesion. Laboratory data showed no abnormalities of routine investigations. Plain X-ray showed no connection with underlying tissue. 

**Figure 2:**
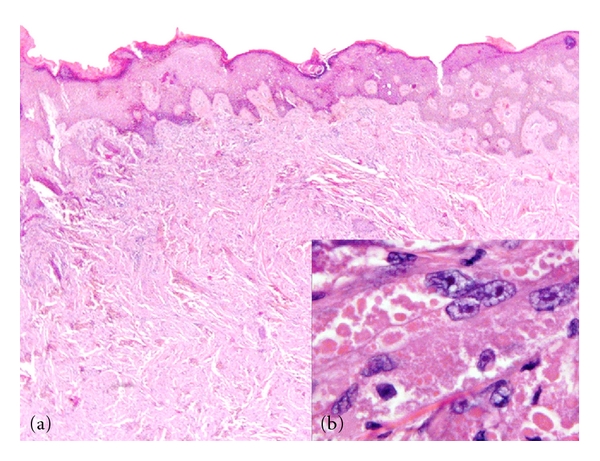
Ill-defined diffuse dermal infiltrate with overlying epidermal acanthosis (a). The tumor cells are large with granular cytoplasm (b) (H&E ×100 and ×1000).

Histologically, the tumor presented with a poorly circumscribed dermal infiltrate which was separated from the epidermis by a clear zone (Figure 2(a)). The infiltrate was formed of sheets, fascicles, and groups of large cells with small rounded nuclei and an eosinophilic granular cytoplasm without observation of significant atypical features in any case (Figure 2(b)).

**Figure 3:**
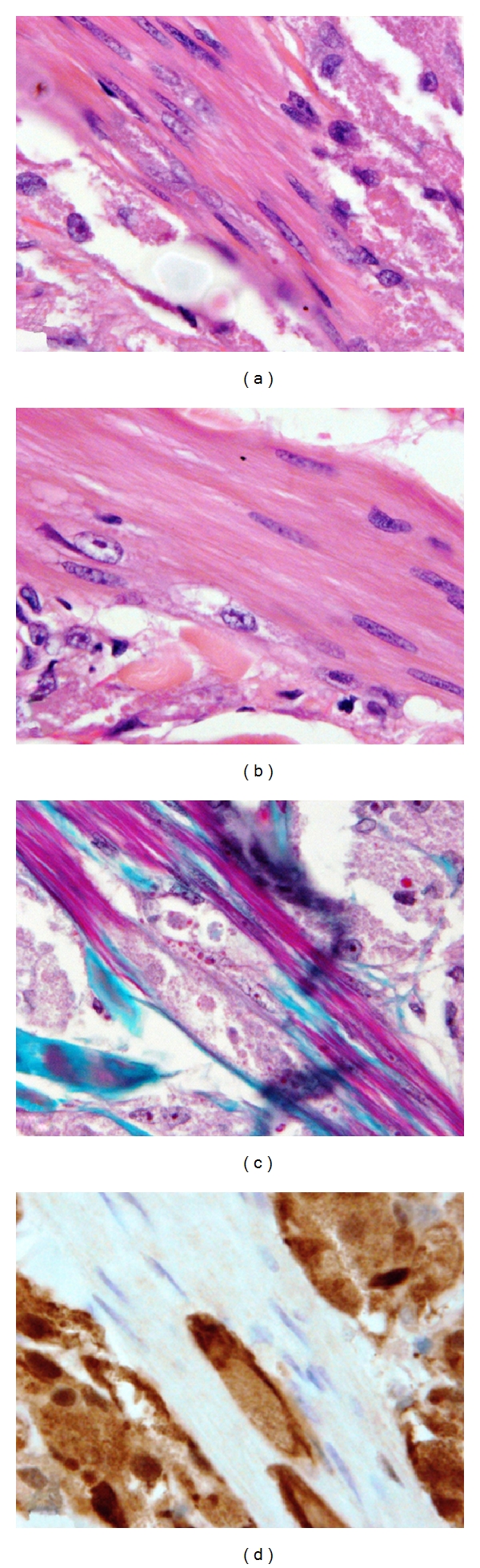
The erector pili muscle is characteristically infiltrated by the tumor cells: H&E (a and b), Masson Trichrome (c), and S100 stain (d) (×1000).

**Figure 4:**
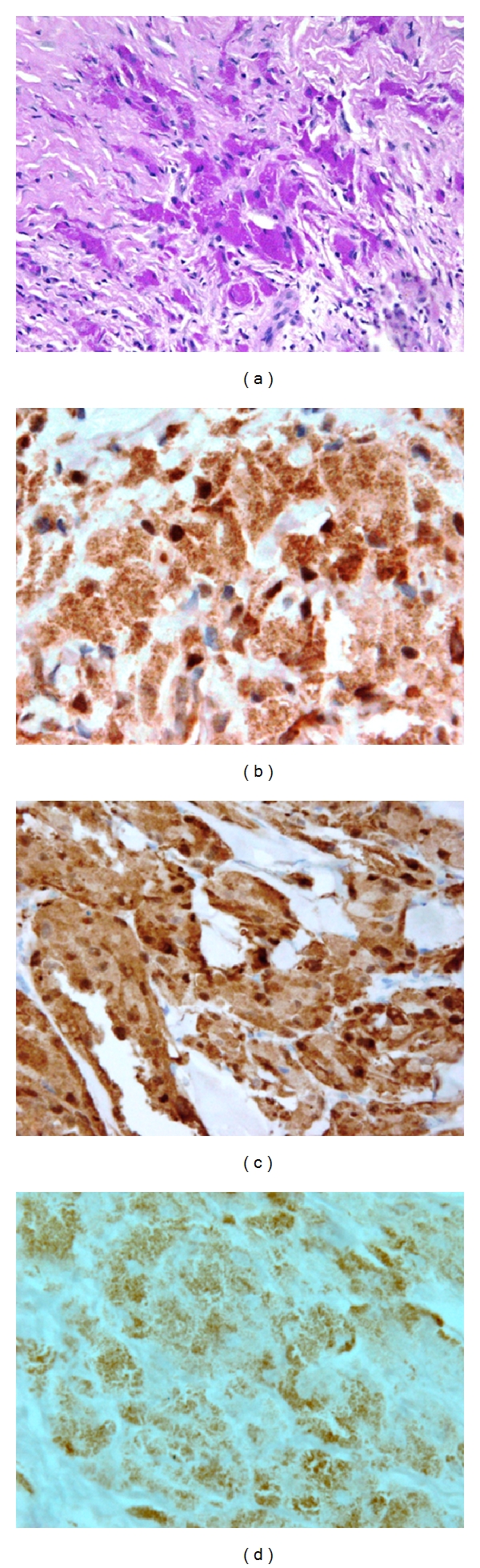
The tumor cells show PAS-positive cytoplasmic granules (a), strong positive immunostaining for S100 (b), and NSE (c) but weakly stained with CD68 (d) (×1000).

The epidermis showed mild to moderate acanthosis, and pseudoepitheliomatous hyperplasia was seen in 3 cases. In two cases the infiltrate was extended into the subcutis. The erector pili muscles showed characteristic infiltration by tumor cells in all cases (Figure 3). PAS stain showed positive staining of the cytoplasmic granules in all cases; the staining was strongly positive in 10 cases (Figure 4(a)) and weak positive in 4 cases. 

Immunohistochemical study showed strong positive staining for S-100 (Figure 4(b)) and NSE (Figure 4(c)) in all cases (100%). Positive immunoreactivity was observed also with Vimentin in 7 cases (50%) and CD68 in 5 cases (35.7%); the staining was mostly strong with Vimentin but it was weak with CD68 (Figure 4(d)). Other markers including cytokeratin, EMA, Desmin, and HMB-45 were negative. Clinical data, histological, and immunohistochemical staining results are summarized in (Table 1).

All patients were referred to surgery department for total surgical excision. Follow-up data were available for only 3 patients who showed no recurrence of the lesion after one year of total excision. The histological and immunohistochemical features of the ulcerative area were similar to that of the borders with the exception of epidermal changes which showed loss of the epidermis with scale-crust formation and the dermal changes which showed increased number of thin-walled capillaries and dense inflammatory cells in the upper dermis.

## 4. Discussion 

The clinical presentation of cutaneous GCT is mostly nonspecific and hardly suspected. Generally it is presented as a solitary asymptomatic nodule, less than 3 cm in size, pink in color, hard in consistency and usually reveals an intact overlying epithelium [6]. The diagnosis of GCT is mostly based on the histological findings and confirmed by immunohistochemical staining which usually shows positive staining for S-100 and NSE [8]. The tumor also expresscs Vimentin, PGP9.5, NKI/C3, and CD68 while some markers such as Inhibin-*α*, Calretinin, Galectin-3, and HBME show varying rates of staining [9]. 

Ulcerative GCT is a rare variant which is not fully studied in the literature, and there was no previous description for the clinical characteristics of this ulcer in addition to the histological and immunohistochemical features of such lesion. The main clinical challenge in ulcerative GCT is the resemblance to infectious granulomatous ulcers especially cutaneous leishmaniasis and tuberculosis in addition to the malignant neoplasms especially squamous cell carcinoma and cutaneous lymphoma. 

The ulcer of cutaneous leishmaniasis usually overlay a large red nodule with formation of central crust. Superficial softness and the volcano sign are important diagnostic features for leishmaniasis ulcer; it is felt soft and slightly mobile over the underlying dermis with indurated firm base, but never hard, and the margin characteristically slopes upwards smoothly, giving the appearance of flattened volcano [10]. The ulcer usually persists for 3–6 months and starts to regress within 5–12 months leaving a sharply demarcated, irregular, cribriform scar [11]. 

In lupus vulgaris, ulceration is uncommon presentation and usually overlay a solitary reddish-brown, flat, soft, or gelatinous plaque with gyrate or discoid shape. Central necrosis occurs which is overlay by a crust. Deep tissue infiltration may occur with cartilage invasion and subsequent contractures or deformities. The lesion is common in adults with female predilection and mostly occurs on the head and neck, and next in frequency are the extremities [12]. The ulcer may exhibit a large size with purulent discharge and foul smelling [13]. Moreover, the ulcer of lupus vulgaris may be due to malignant transformation especially when it shows persistence progression, large size, and lack of response to antituberculous drugs [14]. 

Squamous cell carcinoma (SCC) is an important differential diagnosis for ulcerative GCT especially for the lesions which are located on sun-exposed areas. The nodulo-ulcerative lesion is considered the commonest form of SCC while Marjolin's ulcer which is developed in relation to certain kinds of injury such as burns, scars, and long-standing sores is less frequent [15]. The ulcer of SCC is challengeable because it feels firm with indurated base, usually extended beyond the visible margin of the lesion, and it shows an indurated margin with purulent, exuding surface that bleeds easily. The outline may be rounded, but is often irregular [16]. 

In malignant lymphomas, cutaneous ulceration is usually rare and associated with an aggressive course with poor prognosis. It can occur with a variety of lymphomas, and frequently the ulcers are multiple, necrotic, infected, and placed on tumors [17]. Shah described an ulcer of malignant lymphoma in a 55-year-old man which was located on the upper back. The ulcer was characterized by large size (6 × 7 cm^2^), indurated margin, yellowish-black slough, and foul-smelling discharge, and it was associated with multiple small firm swellings in the surrounding area in addition to enlargement of supraclavicular nodes [18]. 

In this study, we described the clinical features of ulcerative GCT as a large, asymptomatic, nontender rounded, or oval solitary ulcer. It shows indurated base (firm to hard) which extended beyond the surface, necrotic floor (dry in acral location and exudated in genital area), elevated border, and normal margin. The tumor is characterized by slowly progressive course and ulceration usually occurred after a long duration (more than 1.5 years). It is more located on the extremities with male predilection and usually affected young adults (2nd and 3rd decades). These criteria may help to suspect the clinical diagnosis of GCT in ulcerative lesions and to facilitate the clinical differentiation from other ulcerative lesions.

Compared with nonulcerative forms of cutaneous GCT, Ayadi et al. [19] reported that GCT presented clinically as nodular (55.5%) or polypoid lesion (45.5%), often lonely, equal in both sexes (male to female ratio 1 : 1.25); median age was 33.9 years including children, more located on the head and neck with a mean size 1.15 cm and smooth or warty surface. Apisarnthanarax also reported the clinical features of 16 patients with GCT with an average age incidence of 39 years, a greater frequency among Negroes (69%) and females (62.5%). It was presented clinically with asymptomatic solitary mass in 75%, multiple in 25%, and 84% of lesions were located on the skin [20]. 

Although the location of GCT seems to be nonspecific, the oral cavity was reported as the commonest location for GCT followed by the extremities, back, trunk, cheek, and lastly the pubic region [21]. GCT of external genitalia and perineum was described in both males [22, 23] and females [24]. While involvement of the vulva was more reported in the literature [25], anogenital ulcers in this study were only observed in males. 

Histologically, our cases were consistent with classically reported cases which show round or polygonal cells with abundant pale eosinophilic granular cytoplasm and small eccentric nucleus. The cells have indistinct borders and usually arranged in fascicles or sheets. The epidermis usually shows acanthosis and occasionally pseudoepitheliomatous hyperplasia [20]. There was absence of any histological signs that increase the likelihood of malignancy such as mitotic figures, cellular and nuclear pleomorphism, necrosis, wide cellular sheets, spindle-cell structure, and metastasis [26].

An important feature in all cases was the marked infiltration of erector pili muscles with granular cells. Infiltration of skeletal muscle is a common feature of GCT involving the squamous mucosa; in such areas, regenerating and degenerating muscle fibers are entrapped among fascicles of tumor cells [27]. 

The immunostaining in our cases showed strong positive reactivity with S100 and NSE in all cases (100%) while Vimentin showed 50% positivity and CD68 showed positivity in 35.7%. Compared with nonulcerative forms, Ayadi et al. found that tumor cells in nodular lesions were positive for S100 in 100%, Vimentin in 90%, and NSE in 80% while they were negative for cytokeratin and Anti-Mutant Nucleophosmin (AML) [19]. 

The pattern in both variants is more consistent with Schwann cell origin for nongingival GCT which express diffuse cytoplasmic and nuclear staining for S100 protein [28]. This may replace the initial theory which suggested that the tumor cells are derived from muscle cells [1]. The neural histogenetic origin of GCT was also proposed after immunohistochemical analysis of 15 oral lesion by Rejas et al. who found a positive staining for S-100, P75, NSE, and CD68 while other markers including Ki-67, Synaptophysin, HHF-35, SMA, EMA, Chromogranin, Progesterone, Androgen, and Estrogen were negative [29].

Although there was strong staining for CD68 in both GCT and schwannomas in previous studies which strengthen the histogenetic relationship between GCT and Schwann cells [30], we couldnot observe such relation in our cases which expressed weak positive staining in only 35.7% of cases. This may suggest another neural differentiation of such variant but more immunohistochemical studies are required to explain these changes.

Our results denote that ulcerative form of GCT may form a clinical diagnostic challenge but clinical suspicion could be considered in long standing, asymptomatic solitary ulcer which is located on the extremities in a young male. 

## 5. Conclusion 

To our knowledge, this is the first study that discusses the clinicopathological and immunohistochemical features of ulcerative form of GCT. Although it is uncommon variant, we recommend it to be considered in the differential diagnosis of solitary large ulcerative lesions especially those located on the extremities or genital region. 

## Figures and Tables

**Table 1:** The clinicopathological and immunohistochemical features of ulcerative GCT.

Clinical data	
(i) Age (years)	
Range	17–42 y.
Mean ± SD	31.5 ± 7.42

(ii) Sex	
Males/females	11/3
Ratio	3.6 : 1

(iii) Duration of the lesion (months)	
Range	23–51 m.
Mean ± SD	37.2 ± 8.32

(iv) Time of 2ry ulceration (months)	
Range	19–41 m.
Mean ± SD	31.5 ± 6.71

(v) Location	
Extremities	12 (5 arm, 2 forearm, 3 foot, 1 leg and 1 thigh)
Anogenital	1 scrotum and 1 perineum

(vi) Size of the base (cm)	
Range	3.8 × 3.5–5.2 × 5.1
Average	4.1 × 3.9

Histological features	

(I) Epidermal changes	Acanthosis: 11
	Pseudoepitheliomatous hyperplasia: 3

(ii) Cellular infiltrate	Dermal: 12
	Dermal and subcutaneous: 2

(iii) Appendageal infiltrate	Muscle infiltrate: 14

(iv) PAS stain	Strong positive: 10
	Weak positive: 4

Immunohistochemical features	

(i) Positive staining	S100 and NSE: 14 (all strong)
	Vimentin: 7 (6 strong–1 weak)
	CD68 : 5 (all weak)

(ii) Negative staining	Desmin, HMB45, EMA and CK
